# Identification and Characterisation of a pH-stable GFP

**DOI:** 10.1038/srep28166

**Published:** 2016-06-21

**Authors:** Tania Michelle Roberts, Fabian Rudolf, Andreas Meyer, Rene Pellaux, Ellis Whitehead, Sven Panke, Martin Held

**Affiliations:** 1 ETH Zurich D-BSSE, Mattenstr. 26, 4058 Basel, Switzerland; 2 Swiss Institute of Bioinformatics, Mattenstr. 26, 4058 Basel, Switzerland

## Abstract

Green fluorescent proteins (GFPs) are invaluable tools for modern cell biology. Even though many properties of GFP have been successfully engineered, a GFP retaining brightness at low pH has not emerged. This limits the use of GFP in quantitative studies performed in fluctuating or acidic conditions. We report the engineering and characterisation of tandem dimer GFP (pH-tdGFP), a bright and stable GFP that can be efficiently excited and maintains its fluorescence properties in acidic conditions. Therefore, pH-tdGFP could act as a quantitative marker for cellular processes that occur at low pH, such as endocytosis, autophagy or starvation.

Fluorescent proteins are important markers for qualitative and quantitative analysis of biological processes. GFP and its derivatives, gave rise to a whole host of photostable, fast-folding, and bright fluorescent proteins which have been exploited in a diverse array of assays^1^,^2^,^3^. Most often, the different GFPs have been used as biomarkers in which the localisation of tagged proteins or their expression pattern can be monitored *in vivo*^4^. Alternatively, fluorescent assays rely on the ability of fluorescent proteins to report a change in conditions by increasing or decreasing fluorescence intensity. For example, GFP derivatives have been exploited as biosensors that enable quantification of pH changes, the presence of specific ions, reactive oxygen species, and redox state^5^.

In order to perform meaningful quantification of protein localisation changes or differential expression pattern in a cell or a cell population, it is important that the fluorescence intensity of a biomarker remain constant in changing cellular environments. Although GFPs are insensitive to changes in a variety of cellular conditions one issue that remains is their pH sensitivity as the fluorescence significantly decreases under acidic conditions. The pH sensitivity of all GFPs (excited at 488 nm) is attributed to the protonation of the electron-rich, light-absorbing part of the chromophore, which occurs at sub-physiological pH^2^. This sensitivity prevents the use of GFP as a quantitative tool for monitoring acidic intracellular compartments such as lysosomes and vacuoles, organelles involved in the fundamental processes of receptor-mediated endocytosis and autophagy or localisation changes under changing cytoplasmic pH conditions. Therefore, a pH insensitive GFP variant with a stable signal independent of the pH of its cellular target would be a great addition to the repertoire of available GFPs.

Here, we present the development of a GFP variant that could be used as a quantitative biomarker independent of changes in intracellular pH. We performed a directed protein evolution experiment aimed to identify a derivative of superfolder GFP (sfGFP) whose fluorescence remains stable at physiologically relevant pH values^6^. Two concurrent mutations, N149Y and Q204H, resulted in the greatest increase in pH and environmental stability. However, as both amino acids lie within the dimer interface of GFP and led to the dimerisation of the variant, the resulting protein is rendered impractical as a tag for *in vivo* studies. To circumvent this, we generated an intradimerising construct of two sfGFPs containing N149Y and Q204H, separated by a flexible linker, termed pH-tdGFP (pH-stable tandem dimer GFP). pH-tdGFP behaves as a monomer *in vivo* and *in vitro*, performs as predicted for *in vivo* applications at acidic pH and is more stable than sfGFP over the *in vitro*-tested pH range (3.75 to 8.50).

## Results

### Identification of a pH-stable variant of GFP

**Figure 1:**
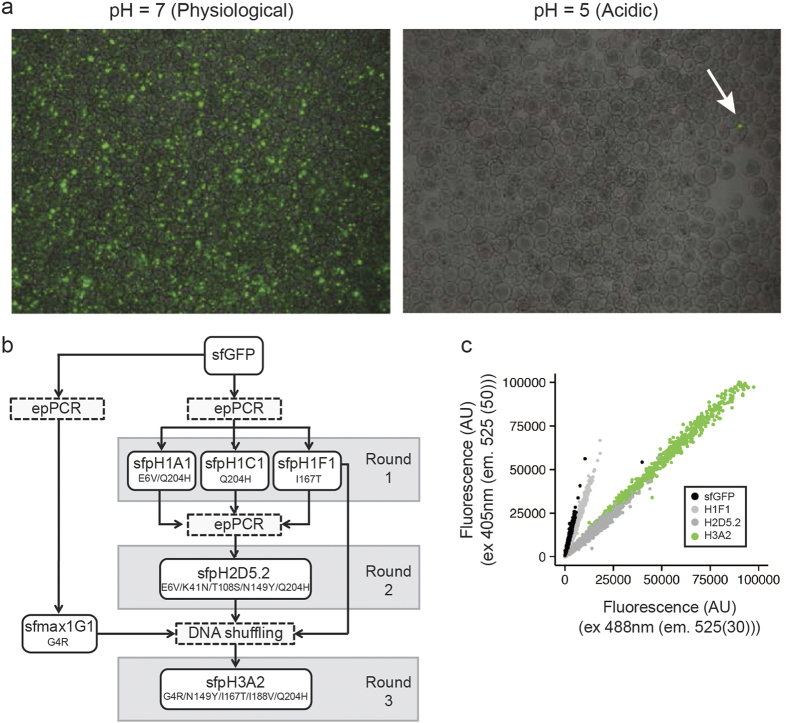
Screening for a pH-stable GFP variant. (**a**) Comparison of the fluorescence of nLR-encapsulated cells expressing the GFP library in physiological versus acidic pH. Arrow indicates a potential pH-stable variant. (**b**) Schematic representation of rounds of mutation and screening to identify a pH-stable GFP variant. (**c**) Comparison of the ratio of fluorescence intensities at pH 5 of indicated variants at 405 nm and 488 nm with constant emission (525 nm).

To identify a pH-stable GFP variant we altered the cytosolic pH of *E. coli* (JM101) cells expressing sfGFP variants by incubation in acetate buffer, pH 5^7^. In total, we performed three rounds of diversification, enrichment and selection of fluorescent clones (Fig. 1a). For the initial two screening rounds, we diversified our library using error-prone PCR with 3–4 base changes per gene. In the last round, we used DNA shuffling of the best candidates from round 1 and 2 (Fig. 1b). In each round we enriched the clones using alginate-based nanolitre reactors (nLRs)^8^. Each nLR was seeded with 2–3 recombinant cells and the cells were allowed to grow until the generated microcolonies consisted of about 1,000 cells. The nLRs were then incubated in acetate buffer (pH 5) to lower the intracellular pH and sorted based on fluorescence intensity (ex 488 nm, em 515(20) nm) using a particle sorter (COPAS)^9^. The 0.1% of nLRs containing the brightest microcolonies were selected for further analysis. To this end, the nLRs were dissolved using citrate buffer solution and the cells were reencapsulated such that a single variant would be present per bead. The screening process was repeated for these single variant nLRs and the 0.01% brightest nLRs were selected. The resulting variants were isolated, their pH stability was confirmed by incubating cells in acetate buffer (pH 5) and the sequence of the confirmed hits was determined. The most pH-stable variants were then used for the next round of diversification.

The first round of screening yielded seven hits, encoding three variants with improved pH stability; A1 (E6V, Q204H), F1 (I167T), and C1 (Q204H). These three variants were pooled and used as the parents for the second round of diversification. The second round of screening identified nine variants, encoding two variants with improved pH stability; D5.1 (E6V, Q69L, Q204H) and D5.2 (E6V, L41N, T108S, N149Y Q204H). The best variant, D5.2, was then shuffled together with variant F1 (I167T) and a mutant with increased expression level (sfmax1G1; sfGFP-G4R) using a low-fidelity polymerase. This final round of screening lead to the identification of a highly pH-stable protein (sfGFP-G4R, N149Y, I167T, I188V, Q204H).

To validate that our screen lead to a pH stability improvement, we tested the change in excitation ratio between the protonated (405 nm) and deprotonated (488 nm) state (Supplementary Fig. 1). We therefore examined the most stable clone identified in each round of screening using flow cytometry (Fig. 1c). A large shift was detectable in the second round, whereas the third round increased the fluorescence intensity but not the pH stability and only a slight shift occurred in the initial round. We therefore reasoned, that the screen was successful and that the mutations most responsible for the pH stability were acquired in the second round.

### Analysis of the mutations resulting in pH stability

In order to confirm our findings and determine the residues required for pH stability we examined various combinations of mutations beginning with N149Y and Q204H as the concomitant appearance of these two mutations in the population resulted in a significant increase in fluorescence intensity ratio at 488 nm vs. 405 nm at pH 5 and apparent pH stability in the second screening round (Fig. 1c, Supplementary Table 1). Moreover, these two mutations were the only ones kept between round two and three as three likely neutral mutations (E6V, K41N, T108S) were lost and two potentially beneficial mutations (I167T, I188V) were gained. The expression capacity mutation G4R is not included in subsequent evaluations of pH stability as the beneficial effect seems to be context specific.

**Figure 2:**
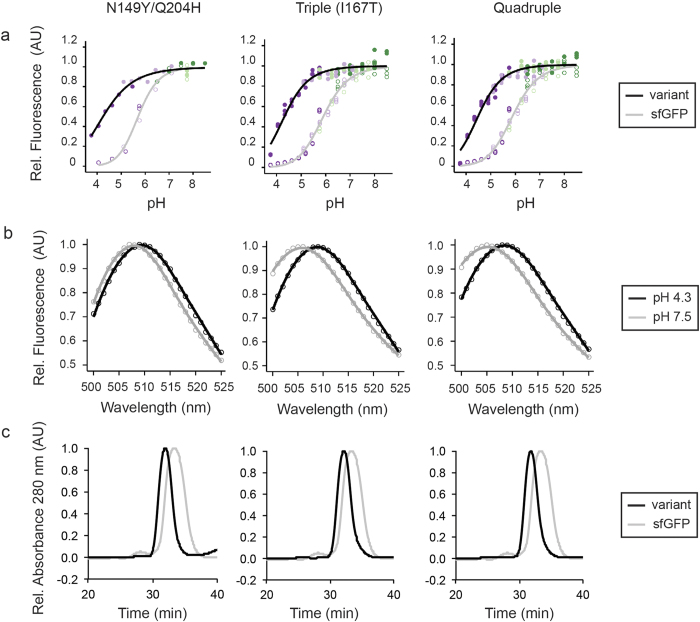
Characterisation of pH-stable GFP variants. (**a**) Purified GFPs were diluted, in quadruplicate, into buffers ranging in pH from 3.75–8.50 and fluorescence was measured (ex 488 nm, em 525 (5) nm). Lines represent the trend of the median fluorescence for the measured pH values. Filled and open circles indicated fluorescence values at each pH for the indicated variants and sfGFP respectively; colours indicate the buffer used (acetate (dark purple), MES (light purple), PIPES (light green), HEPES (dark green)). sfGFP curves on each graph are from the same date and plate as the indicated variant. (**b**) Emission scans of GFP variants excited at 488 nm were performed at pH 4.3 (black) and 7.5 (grey). Scans are plotted relative to the peak of each scan. (**c**) Oligomeric state of purified GFP variants was examined by size exclusion chromatography. For each graph the variants are indicated and sfGFP is shown in grey as a reference. Triple (167T) denotes N149Y/Q204H/I167T; quadruple denotes N149Y/Q204H/I167T/I188V.

To test the effects of those mutations more carefully, we performed a pH titration between pH 3.75 to 8.50 using indicated purified GFP proteins in various buffers selected to maintain the ionic strength. We chose this range as pH 3.75 was the lowest possible pH at which the proteins remained in solution and a pH above 8.50 is outside the physiological range. Both single mutants Q204H and N149Y exhibited a modest increase in pH stability relative to sfGFP (Supplementary Fig. 2). However, the combination of Q204H with N149Y led to a highly pH-stable variant (Fig. 2a). Addition of I167T seemed beneficial as it led to an increase in pH stability, while addition of I188V was detrimental. This effect was also seen in the quadruple mutant where I188V was added to the I167T triple mutant (the fitted data from the different pH titration experiments are summarised in Supplementary Table 2). Taken together, the concomitant introduction of Q204H and N149Y is required for an increase in pH stability. Addition of I167T increases the stability further, however the I167T single mutation is unable to lock the chromophore in the deprotoned state as shown in the initial characterisation (Fig. 1c).

One important characteristic of a pH-stable variant is, that its spectral properties remain unchanged at different pH conditions. To this end, we acquired excitation and emission spectra at pH 4.3 and 7.5. The emission spectra of variants containing mutations in N149Y, Q204H and I188V exhibited only a minor shift between high and low pH. However, the emission maxima for all variants carrying the I167T mutation was dependent on pH (Fig. 2b, Supplementary Fig. 3). Furthermore, the introduction of the mutation I167T resulted in a shift of the emission maxima from 510 nm to 503 nm at pH 7.5 as reported earlier^1^ (Supplementary Fig. 4). As expected from the flow cytometry data, the excitation spectra of all variants were stable at high and low pH in contrast to that of sfGFP (Supplementary Fig. 5).

### Mutations increasing pH stability result in dimerisation of sfGFP

Examination of the crystal structure of wild-type GFP revealed that N149 and Q204 are surfaced exposed in monomeric GFP and lie within the dimer interface of dimeric GFP^10^. As such, we wanted to examine whether mutation of these residues led to a dimeric sfGFP variant. We used size exclusion chromatography and semi-native PAGE to examine the oligomeric state of our variants and found that all were shifted to a higher molecular weight (Fig. 2c, Supplementary Figs 6 and 7)^11^. This indicates that while sfGFP appears to be primarily present as a monomer, all mutants tested (N149Y, Q204H, N149Y/Q204H, N149Y/Q204H/I167T, N148Y/Q204H/I188V, and N149Y/Q204H/I167T/I188V) are present primarily as dimer.

As the mutations that lead to increased pH stability also lead to dimerisation, we wondered if dimerisation itself was the cause of the pH stability. To examine this, we reversed the so-called monomerising mutation at position 206. Substitution of alanine 206 with a valine or lysine is believed to result in the monomerisation of various fluorescent proteins, including sfGFP^2^. As such, we introduced an alanine at position 206 in order to dimerise sfGFP without additional substitutions. Interestingly, we found that the pH stability of the V206A variant was similar to sfGFP, suggesting that other factors are important for the pH stability (Supplementary Fig. 8).

Oligomerisation of proteins is associated with increased stability^12^. We therefore measured the guanidinium hydrochloride induced equilibrium of unfolding after five days of incubation. N149Y and Q204H both increased the equilibrium concentration of unfolding, while their combination was not additive and remained at the value for Q204H. Addition of I167T or I188V to create a triple mutant, destabilised the protein to even lower values than the original sfGFP and the quadruple mutant was further destabilised suggesting mutations to I167 or I188, which lie in the protein core, are detrimental for protein stability (Supplementary Fig. 9).

### Development of a pH-stable GFP variant for use as biomarker

**Figure 3:**
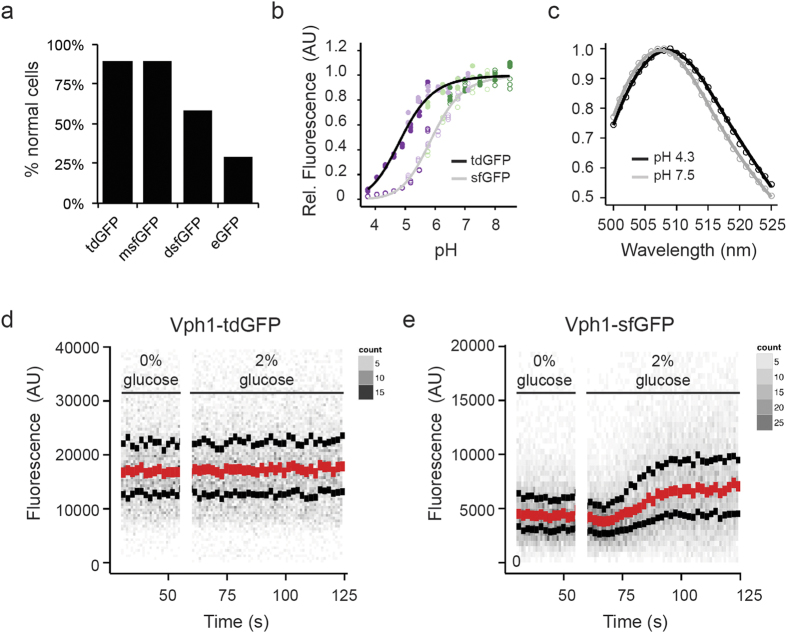
Characterisation of pH-tdGFP as a biomarker. (**a**) Intracellular oligomerisation of pH-tdGFP was examined. Indicated GFPs fused to a C-term portion of cytochrome p450 were expressed in HEK293 cells. GFPs that oligomerise cause ‘whorl’ structures in the ER. The number of cells expressing GFP were counted and those with whorls were considered abnormal. msfGFP (monomeric sfGFP; Lys at position 206), dsfGFP (dimeric sfGFP; Ala at position 206) (**b**) Purified pH-tdGFP was diluted into buffers ranging in pH from 3.75–8.50 and fluorescence was measured (ex 488 nm, em 525 (5) nm). sfGFP (pKa = 5.9) is present as a reference. Colours indicate the buffer used (acetate (dark purple), MES (light purple), PIPES (light green), HEPES (dark green)). (**c**) Emission scans of pH-tdGFP variants excited at 488 nm were performed at pH 4.3 (black) and 7.5 (grey). Curves are plotted relative to the peak of each scan. Yeast cells expressing pH-tdGFP-tagged Vph1 (**d**) or sfGFP tagged Vph1 (**e**) were incubated in media +/− 2% glucose to examine the *in vivo* pH stability of pH-tdGFP. In media lacking glucose the pH drops to ~5.5 and rises to ~7.5 upon readdition of the glucose. Red dots indicate median with the upper and lower black dots indicating the quartiles. Grey dots indicate the number of cells as indicated by the heat maps.

A dimeric GFP variant is not useful as a biological tool as it can artificially dimerise tagged proteins. In order to develop a useful monomeric, pH-stable GFP we generated a tandem dimer; a strategy successfully applied for tdTomato, an apparently monomeric red FP^13^. In order to create a pH-stable variant that retains the spectral and thermodynamic qualities of sfGFP, we decided to incorporate only N149Y and Q204H mutations into the tandem dimer construct. To create a monomeric GFP variant, we created a construct comprised of two sfGFP-N149Y/Q204H separated by a flexible 25 amino acid linker, termed pH-stable tandem dimer GFP (pH-tdGFP). This variant should intra-dimerise and therefore result in an effective monomer. To examine the intracellular oligomerisation state of pH-tdGFP, we used the recently described organised smooth ER (OSER) assay^14^. In this assay protein tags of interest are fused to the cytoplasmic end of an endoplasmic reticulum signal anchor protein (cytochrome P450). If the tags dimerise, it artificially dimerises two p450 molecules resulting in restructuring of the ER into OSER ‘whorl’ structures. We expressed pH-tdGFP, monomeric sfGFP (msfGFP; Lys at position 206), dimeric sfGFP (Ala at position 206), and EGFP (enhanced GFP) as fusions in HEK293T cells and counted the percentage of normal cells. At least 50 normal looking cells from two transfections were counted (pH-tdGFP n = 109, 101; msfGFP n = 101, 100; dsfGFP n = 116, 102; EGFP n = 52, 88). We found that pH-tdGFP (90%), behaved similar to msfGFP (90%) in contrast to dimeric GFP (59%) and EGFP (29%). Since pH-tdGFP exhibited a lack of whorl structures similar to that of msfGFP this indicates that it is primarily monomeric in the cell (Fig. 3a, Supplementary Fig. 10a). The monomeric state of pH-tdGFP was further validated by size exclusion chromatography and semi-native PAGE (Supplementary Fig. 10b,c). Both assays revealed that pH-tdGFP behaves as a monomer. Taken together our results indicate that by creating a tandem dimer, pH-tdGFP is present primarily as a monomer by dimerising in *cis* and not in *trans* with another pH-tdGFP molecule.

To further validate that pH-tdGFP is a useful protein tag, we constructed *Saccharomyces cerevisiae* strains in which the cytoplasmically located C-terminus of the ATPase V0 sector protein, Vph1, was endogenously tagged with either sfGFP or pH-tdGFP. We monitored the effect of the pH-tdGFP tag on Vph1 and found that it had little effect on the function, while sfGFP showed a slight impairment (Supplementary Fig. 11). As pH-tdGFP behaves well in the OSER assay and does not interfere with a protein function *in vivo*, we reasoned that it can be employed as useful protein tag even with difficult to tag proteins.

To explore whether pH-tdGFP retained the pH and thermodynamic stability observed for sfGFP-N149Y/Q204H, we measured the fluorescence of purified pH-tdGFP in pH and guanidinium hydrochloride titration experiments. pH-tdGFP was found to retain the pH stability (pKa 4.8) and exhibits an increased protein stability compared to the double mutant (Fig. 3b, Supplementary Fig. 12). Moreover, the emission spectra at high and low pH remains unchanged (Fig. 3c, for easier comparison, we summarised the properties for all the different protein variants in Supplemental Table 3). Taken together, pH-tdGFP is a monomeric protein, that is much more stable than sfGFP at low pH values and possesses an increased protein stability.

To examine its *in vivo* application in conditions where pH changes, we used the starvation response of *Saccharomyces cerevisiae* as a model. The pH of the cytoplasm of yeast is affected by glucose levels and can be manipulated upon rapid switching of media containing and devoid of glucose^15^. When grown on glucose, cells have an intracellular pH of ~7.5. After glucose removal the pH drops over a period of 15 min to pH ~6 and then stabilises. The re-feeding of glucose leads to a fast (~30 s) drop of the cytoplasmic pH to about 5.5 followed by a somewhat slower (~5 min) increase until a pH of 7.5 is reached (>15 min). To analyse whether the fluorescence of pH-tdGFP was sensitive to these intracellular pH fluctuations over time, cells were grown in synthetic complete (SC) media to mid-log phase and then starved for glucose for 30 minutes, at which point glucose was reintroduced. The fluorescence was monitored by flow cytometry. Strikingly, the median cellular fluorescence intensity during glucose removal and re-addition remained constant when pH-tdGFP was used as tag, but cells expressing Vph1-sfGFP exhibited variability in fluorescence (Fig. 3d,e). These results indicate that the fluorescence of pH-tdGFP is, in contrast to sfGFP, stable over a broad range of physiological intracellular pHs from ~5.5 to 8.0.

## Discussion

We describe pH-tdGFP, a GFP variant, whose fluorescence is stable at pH as low as 5.5. We isolated beneficial mutations using an easy, fast, and robust technology for screening of microbial microcolonies. Careful examination of the identified mutations revealed that the concomitant introduction of N149Y and Q204H resulted in a pH-stable GFP but also led to dimerisation of the protein. The correlation between dimerisation and pH stability seems to be specific, as dimerisation by reversal of the monomerising mutation at position 206 did not lead to an increase in pH stability. This suggests that the nature of the dimer may be critical for the observed pH stability, especially as introduction of either N149Y or Q204H alone only led to a moderate increase in pH stability compared to sfGFP. One possibility is that introduction of a single dimerising mutation is not enough to exclude the proton from the local environment of the chromophore as a single interaction would result in a hinge allowing the dimers to expand and contract thus allowing protons to come and go. However, if two amino acids are participating in the dimer at distinct positions they could act as a “lock” that effectively prevents protons from entering, thereby keeping the chromophore in the deprotonated state. It is interesting to note that prolonged exposure of purified N149Y/Q204H GFP to an acidic environment did not alter the fluorescence intensity suggesting that it is possible that the dimerisation leads to the chromophore being trapped in an insulated cavity; however, even though N149 and Q204 face away from the chromophore we cannot rule out the possibility that the environment of the chromophore is altered by mutation of these residues resulting in a structure which can no longer switch between the protonated and deprotonated forms.

Dimerisation of fluorescent protein tags is a quality undesirable for *in vivo* studies. To circumvent this issue we constructed a tandem GFP with each sf GFP harbouring two mutations (N149Y and Q204H) and connected by a flexible linker. We demonstrated that this tandem dimer intra-dimerises in solution creating an effective monomer and that, importantly, *in vivo* studies indicate that proteins tagged with pH-tdGFP can localise normally and retain wild-type function. Using standard assays in *S. cerevisiae*, we demonstrated that pH-tdGFP is insensitive to changes in cytoplasmic pH. We also showed that pH-tdGFP fused to the C-terminal of cytochrome p450 localises normally in HEK293 cells. Furthermore, tandem fluorescent protein tags, such as tdTomato and mCherry-GFP, have been successfully used in a number of studies indicating that pH-tdGFP could be used as a functionally relevant biomarker^16^. Compared to the red pH stable fluorescent proteins such as mCherry and mKOK, GFP matures faster and compared to mTfp1 is more sensitive as the slight red shift of the emission can be collected at a wavelength band where cells typically emit less autofluorescence. Additionally, most labs are well equipped to measure GFP variants. Based on its pH stability and performance as a tag for yeast Vph1 and in HEK293 cells we believe that pH-tdGFP could be a useful tag for monitoring processes occurring in changing pH environment, such as endocytosis or autophagy, that involve trafficking through acidic cellular compartments or quantification of protein localisation and expression under starvation conditions.

## Materials and Methods

### Gene diversification and library generation

For the generation of DNA libraries two different diversification methods were used: error-prone PCR (epPCR) and DNA shuffling^17^,^18^. sfGFP was chosen as the parental GFP as it is a robust folding variant and would thus likely tolerate many additional mutations^6^. Details are listed in the Supplementary Methods.

### Encapsulation of *E. coli* in nLRs

A specific number of cells from a pre-calibrated glycerol stock, typically the number required to adjust an average final occupation of 2–3 cells per nLR and provided in a volume of approximately 20 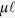, was mixed with 1 m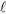 of 0.9% (w/v) aqueous NaCl. The resulting cell suspension was added to 20 m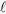 of an alginate solution (4% w/v medium viscosity sodium alginate (Sigma, Buchs, Switzerland) in 0.9% NaCl, autoclaved for 20 min at 121 °C) and vigorously mixed (5 min, room temperature). The mixture was allowed to stand (20 min, room temperature) in order to remove the air bubbles formed during mixing and then processed to droplets by an aerodynamically assisted jetting device (J30, Nisco Engineering AG, Zurich, Switzerland) using a 350 *μ*m diameter nozzle, a flow rate of 0.4 m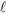 per min, and a pressure drop of 70 mbar (synthesis rate of ~20,000 per second). The droplets were collected in a magnetically stirred beaker filled with 100 m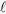 of 100 mM CaCl_2_ and allowed to mature for 15 min, resulting in nLRs of an average diameter of 90 *μ*m (300 pL). nLRs used for selection: The alginate precursor/cell mix was prepared as above but to an average final concentration of 0.3 cells per nLR in a final alginate concentration of 2.7%. The suspension was processed by a laminar jet break-up system (Nisco Engineering AG, Zurich, Switzerland) using a 150 *μ*m diameter nozzle and a flow rate of 3.8 m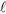/min resulting in a bead-production frequency of 680 Hz. The resulting droplets were collected in a magnetically stirred beaker as above and allowed to mature for 30 min, resulting in nLRs of a diameter of approximately 470 *μ*m (55 nL).

### Proliferation of *E. coli* in nLRs

nLRs were recovered from the hardening solution by sieving (40 *μ*m Falcon sieve, BD, Franklin Lakes, NJ) and aliquots of 2 g of wet nLRs were added to Petri dishes containing 20 m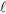 of OB 50 growth medium (per liter of water 4 g yeast extract, 2 g bacto tryptone, 1 g glycerol; 100 mg/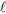 ampicillin, 0.2 mM IPTG) and incubated for up to 14 h at 30 °C. The nLRs were isolated by sieving, washed three times with 50 m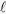 of 10 mM CaCl_2_ and kept at 4 °C until processed further.

### Screening and enrichment

After incubation in growth medium (see above), nLRs were suspended in 10 mM CaCl_2_ solution containing a 200 mM NaAc/HAc buffer adjusted to pH 5, incubated at room temperature for 10 min and then analysed by a COPAS Plus (Union Biometrica, Holliston, MA) fluorescence-assisted particle sorter, which processes particles with a diameter of up to 1 mm, uses laser-light of 488 nm (bandwidth 23 nm) for excitation and records fluorescence at 514 nm (bandwidth 25 nm). Photomultiplier voltages were generally in the range of 320 to 400 V. In a typical primary screen (or enrichment round), libraries were oversampled by approximately a factor of 10 at an analysis rate of 600 nLRs per second. The nLRs displaying the most fluorescence were sorted into a Petri dish using the “enrich” sorting mode of COPAS. In this mode all droplets containing a particle that matches pre-set specifications are selected regardless of the potential presence of a second nLR within the same droplet. After screening, 3 m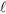 of LB medium were added to the Petri dish, the cells were released into the medium and incubated overnight (30 °C). Next, glycerol stocks were prepared and an aliquot was encapsulated in nLRs for screening and isolation of single clones (see above). In a typical nLR experiment (“selection”), 50,000 nLRs were analysed at a rate of 20 nLRs per second and dispensed into glass-bottom 96-well plates containing 50 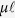 of 10 mM calcium chloride. The sorting was performed in the “pure” mode of COPAS (ensuring only one particle per selected droplet) and all nLRs containing more than one colony were excluded using the COPAS profiler software^9^. The monoclonality of sorted nLRs was also confirmed using an inverted fluorescence microscope (Axio Observer, Zeiss, GFP filter set). A 100 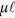 aliquot of growth medium (LB, 100 mg/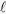 ampicillin) was added to positive wells and the liberated cells were incubated for 14 h at 30 °C, cultivated on LB agar plates (LB, 100 mg/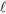 ampicillin, 0.2 mM IPTG), and reisolated.

### Purification of GFP variants

GFPs were expressed from a pKQV5 plasmid (all sfGFP variants) under the control of a tac promoter or from a pJexpress plasmid (pJExpress401-T5-kan-High) under control of the T5 promoter obtained from DNA2.0. pH-tdGFP is a tandem fusion of two yeast codon optimized sfGFP-N149Y/Q204H separated by the amino acid linker ITLGGTGSGSGDEVDGMVSKGEEVIK. The construct was synthesised by Geneart. Expression was done in 50 m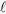 LB containing 100 *μ*g/m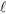 kanamycin and 200 *μ*M IPTG. Cells were harvested, lysed by sonication and purified using an ethanol extraction method^19^. Proteins were stored in PBS. Impurities were observed for pH-tdGFP and so pH-tdGFP was subjected to further purification using centrifugal filter units with a 50 kDa cutoff (Millipore, Billerica, MA). Purity was checked by SDS-PAGE and was determined to be greater than 95% for all purified fluorescent proteins (Supplementary Fig. 13).

### Examination of pH stability

Purified GFP variants were diluted into buffers ranging in pH from 3.75–8.50 in 384-well microtitre plates using a Tecan EVO 200 robotic system. Specifically, sodium acetate buffer (pKa 4.75) ranging from pH 3.75–5.75, MES buffer (pKa 6.1) from pH 5.1–7.1, PIPES buffer (pKa 6.75) from 5.75–7.75 and HEPES buffer (pKa 7.5) from 6.50–8.50. Emission and excitation spectra were subsequently recorded using a Tecan M200 or M1000 plate reader. The obtained data were fitted to the three state logistic model *maxvalue*/(1 + (*pH*/*pK*_*a*_)^*slope*^) using the nls routine in R. The 95% confidence interval of the fit was then predicted using the confint routine.

### Examination of *in vitro* oligomeric state

Purified GFP variants were resuspended in 5x Laemmli buffer containing 0.2% SDS and 12.5 mM EDTA but no *β*-mercaptoethanol. Samples were loaded without heating and separated on 12% SDS-PAGE gels in running buffer containing 0.2% SDS, 150 V for 60 min^11^. Gels were visualised using an LED (470 nm) illuminator (Maestrogen, NV) and raw images recorded using a Nikon D3100. Size exclusion chromatography was performed using a Superdex S200 10/300 GL column (GE Healthcare, Switzerland) and PBS (pH = 7.4) as buffer. Approximately 500 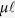 of 1 mg/m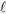 fluorescent protein solution was injected. Retention time was monitored using UV absorbance (280 nm).

### Examination of oligomeric state using the mammalian cell-based OSER assay

HEK293T cells were transfected using a standard protocol^20^ with 2 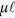 of 2–10 *μ*g/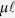 plasmid DNA and were examined 30 h after transfection using an automated Nikon Ti-Eclipse microscope with a 10x/0.45 NA air objective, a 490 nm Cool LED pE-2 excitation light source, a Hamamatsu Orca Flash 4 camera and a 480 (20) nm excitation filter, a 505 nm dichroic mirror and a 530 (11) nm emission filter. Cells with puncta were counted has abnormal cells containing whorls (Supplementary Fig. 14).

### Yeast strains and manipulations

All experiments performed with yeast were with the BY4741 (available from Euroscarf) strain background^21^. Endogenous tagging of Vph1 with either sfGFP or pH-tdGFP was performed using modified pFA6 cassettes (original plasmid pKT0103) and standard yeast manipulation techniques^22^,^23^,^24^, resulting in the yeast strains; VPH1-sfGFP::URA3MX6 (FRY40) and VPH1-pH-tdGFP::URA3MX6 (TRY285). YFP venus in the modified pKT0103 was exchanged with sfGFP and pH-tdGFP (FRP1647) respectively using the PacI/AscI sites present in the original vector. Plasmid FRP1647 is available from Addgene https://www.addgene.org/74322/.

### Flow cytometry of Vph1 tagged yeast

Cells expressing Vph1-sfGFP or Vph1-pH-tdGFP were monitored in a Fortessa flow cytometer with excitation of 488 nm and emission 525 (30) nm to examine GFP fluorescence and excitation of 445 nm and emission 535 (30) nm to monitor changes in cell size via autofluorescence. Yeast strains were grown in YPD or synthetic complete (SC) medium to saturation^24^. The culture was then diluted in SC media containing 2% glucose to exponential phase and initial fluorescence was recorded, cells were harvested by filtration and resuspended in SC media lacking glucose and incubated at 25 °C for 30 min. Fluorescence was recorded in starvation and upon addition of 2% glucose. Experiments were performed at least three times with a minimum of 1,000 cells examined per second.

## Additional Information

**How to cite this article**: Roberts, T. M. *et al.* Identification and Characterisation of a pH-stable GFP. *Sci. Rep.* **6**, 28166; doi: 10.1038/srep28166 (2016).

## Supplementary Material

Supplementary Information
