# Corrigendum: Identification and Characterisation of a pH-stable GFP

**DOI:** 10.1038/srep46976

**Published:** 2018-05-17

**Authors:** Tania Michelle Roberts, Fabian Rudolf, Andreas Meyer, Rene Pellaux, Ellis Whitehead, Sven Panke, Martin Held

Scientific Reports 6: Article number: 2816610.1038/srep28166; published online: 06 21 2016; updated: 05 17 2018

This Article contains errors in the Results section under the subheading ‘Development of a pH-stable GFP variant for use as biomarker’,

“To create a monomeric GFP variant, we created a construct comprised of two sfGFP-N149Y/Q204H separated by a flexible 25 amino acid linker, termed pH-stable tandem dimer GFP (pH-tdGFP).”

should read:

“To create a monomeric GFP variant, we created a construct comprised of two sfGFP-N149Y/Q204H separated by a flexible 22 amino acid linker, termed pH-stable tandem dimer GFP (pH-tdGFP).”

Additionally, in the Materials and Methods section under the subheading ‘Purification of GFP variants’,

“pH-tdGFP is a tandem fusion of two yeast codon optimized sfGFP-N149Y/Q204H separated by the amino acid linker ITLGGTGSGSGDEVDGMVSKGEEVIK.”

should read:

“pH-tdGFP is a tandem fusion of two yeast codon optimized sfGFP-N149Y/Q204H separated by the amino acid linker HGTGSTGSGSSGTASSEDNNMA.”

