# Supplementary material for: Identification and Characterisation of a pH-stable GFP
Source: Sci Rep. 2016 Jun 21;6:28166. doi: 10.1038/srep28166 (PMC4914982; doi:10.1038/srep28166)
Supplement: Supplementary Information [file srep28166-s1.pdf]

Supplementary Material
Identification and Characterisation of a pH-stable GFP

Tania Michelle Roberts[†,1], Fabian Rudolf[†,*,1,2], Andreas Meyer[1,‡], Rene Pellaux[1,‡], Ellis Whitehead[1,2], Sven Panke[1] and Martin Held[*,1]

[1]ETH Zurich D-BSSE, Mattenstr. 26, 4058 Basel, CH
[2]Swiss Institute of Bioinformatics, Mattenstr. 26, 4058 Basel, CH
[†]these authors contributed equally to this manuscript
[*]Correspondence to fabian.rudolf@bsse.ethz.ch or martin.held@bsse.ethz.ch
[‡]present address: FGen GmbH, Hochbergerstr. 60C, 4057 Basel, CH

Supplementary Methods

Strains and plasmids

E. coli JM101 (glnV44 thi-1 Δ(lac-proAB) F'[lacIqZΔM15 traD36 proAB+]) was used for cloning and expression of the DNA libraries as well as for the synthesis of His-tagged wild-type and variant fluorescent proteins. The vector pKQV5 (Ptac, lacIq, ampicillin resistance gene, pBR322 origin of replication) was used for expression of recombinant genes encoding fluorescent proteins in all experiments with *E. coli.* Plasmid pKQV5 was constructed from pKQV41 by eliminating the AfeI fragment containing the tetracycline resistance gene, leaving only the bla resistance gene in the vector. Genes were then cloned as EcoRI/HindIII fragments behind the tac promoter and expression was induced by the addition of 0.2 mM isopropyl-β-D-thiogalactopyranosid (IPTG). N149Y, Q204H, I167T, I188V and variants thereof were introduced to the sfGFP-pKQV5 construct using site directed mutagenesis. The pH-tdGFP OSER plasmid (TRP20) was constructed by inserting pH-tdGFP into the PacI/AscI sites of FRP1193 a plasmid generated from pEGFP-N1-CytERM-EGFP. FRP1193 was constructed by modifying pEGFP-N1-CytERM-EGFP by PCR with phosphorylated primers (OSER fw: GGCGCGCCTAAAGCGGCCgcggccgcgactc, OSER rv: GGTTAATTAAATcccgggcccgcgctaccgtc to generate an plasmid termed "empty OSER" in which ligation of any gene into the PacI/AscI sites leads to a fusion with the P450 domain.

Diversification and library generation

Error-prone PCR (epPCR) was performed with primers that bind approximately 100 bp upstream of the EcoRI and downstream of the HindIII restriction site, respectively, on pKQV5 (KQ-up, 5'-GAGTTCGGCATGGGGTCAGGTGGG-3'; KQ-down, 5'-GCGCCGACATCATAACGGTTCTGGC-3'). The amplification reaction was performed using Taq DNA polymerase and standard buffer (New England Biolabs) supplemented with 2.5 mM $MgCl_2$ and 0.1 mM $MnCl_2$. The nucleotide concentration was 0.2 mM for GTP and ATP and 1 mM for CTP and TTP. Cycling was performed in an Eppendorf Mastercycler with initial denaturation at 95°C for 5 min and 30 cycles of denaturation at 95°C (30 sec), annealing at 55°C (30 sec), and elongation at 72°C (120 sec). A final incubation at 72°C for 10 min allowed finishing of incomplete amplification products. For DNA shuffling, a standard PCR reaction with Taq DNA polymerase and the primers described above was used for the synthesis of the starting sequences. After purification of the PCR products, the DNA was digested with DNAseI in 50 mM Tris-HCl buffer pH 7.5, 10 mM $MnCl_2$ in a total volume of 50 $\mu\ell$. To do so, the reaction mixture was pre-incubated in an Eppendorf Mastercycler at 15°C for 3 min, and then 5 $\mu\ell$ of DNAseI solution (0.2U $\mu\ell$, New England Biolabs) were added. After incubation for 2 min at 15°C the DNAse was deactivated by raising the temperature to 98°C for 10 min. The DNA fragments were subsequently purified with a nucleotide removal kit (QIAquick, Qiagen). Primerless assembly was performed with iProof DNA polymerase (Bio-Rad) using the standard buffer and 1 mM dNTPs each. Thermal cycling included denaturation at 98°C (30 sec), annealing at 45°C +0.1°C cycle 1 (30 sec), and elongation at 72°C (15 sec +1 sec cycle 1) for 40 cycles. After the last cycle

incubation at 72°C for 10 min was done to allow finishing of incomplete amplification products. Amplification of assembled genes was performed by PCR with iProof DNA polymerase (Bio-Rad) with 1 $\mu\ell$ of assembly product (diluted 1:100 with water) as the template and the standard protocol given. Thermal cycling included an initial denaturation step at 98°C for 5 min, and 30 amplification cycles of 98°C for 30 sec, 55°C for 30 sec, and 72°C for 15 sec. After the last cycle incubation at 72°C for 10 min was done to allow finishing of incomplete amplification products. DNA libraries were generated by cloning the PCR products from the diversification steps as EcoRI/HindIII fragments into pKQV5. The ligation products were electroporated into *E. coli* JM101 made electrocompetent according to standard protocols. After the incubation step in SOC medium for 1 h an aliquot of the transformed cells was plated-out on agar plates to determine the library size. The residual reaction mixture was used to inoculate a 5 mℓ LB culture containing ampicillin (100 mg/ℓ) for the selection of cells containing a plasmid from the library and incubated o/n at 37°C. From this culture glycerol stocks were prepared and stored at -80°C until further use. Libraries typically contained 100,000-200,000 individual members and were obtained directly from the glycerol stocks in the subsequent screening experiments.

Initial characterisation of selected proteins

The pH stability of fluorescent proteins in the selected strains were initially characterised in a whole cell assay. Strains were grown overnight in 5 mℓ LB medium containing 0.2 mM IPTG, harvested by centrifugation, and resuspended in 100 μM carbonyl cyanide *m*-chlorophenylhydrazone (CCCP; Sigma Aldrich) in water in order to uncouple the transmembrane proton-gradient. An aliquot of 100 $\mu\ell$ of cells was then diluted into an equal volume of 50 mM citrate-phosphate buffer with pH values in the range of 4.0 to 7.5. The fluorescence was recorded with a Victor3 Multilabel plate reader (Perkin Elmer) with an excitation filter of 485 (15) nm and an emission filter of 530 (25) nm. Strains that showed increased pH-stability were selected, cell free extracts were prepared by glass bead grinding, and the fluorescence was measured in analogy to the whole cell assays but without addition of the uncoupler CCCP. The genes of fluorescent protein variants with confirmed lower pKa values were isolated and sequenced to identify amino acid substitutions.

Examination of stability to denaturation by Guanidinium hydrochloride

Purified GFP variants were diluted into buffers ranging in guanidinium hydrochloride (GuHCl) concentrations from 0-7.5 M in 384-well microtitre plates using a Tecan EVO 200 robotic system. Fluorescent measurements were performed using a 488 nm laser and recorded periodically over a 5 day period using a Tecan M200 or M1000 plate reader. After 5 days, a state was reached where the fluorescence measurements was stable for all constructs and that time-point was used to calculate the standard free energy of denaturation, $\Delta G° = -RT \ln K$, where R is the gas constant, T is the absolute temperature and K is the equilibrium constant. K was calculated from the experimental data with the following equation K = fluorescence[xM GuHCl]/(fluorescence[0M GuHCl]

- fluorescence[xM GuHCl]). The stability of the various GFP variants was subsequently estimated using the equation $\Delta G° = \Delta G(H_2O) - m[GuHCl])$. $\Delta G°$ is an estimate of the conformational stability of a protein that assumes that the linear dependence continues to infinite dilution of denaturant, and m is a measure of the dependence of ΔG on GuHCl concentration.

Calcium Sensitivity and Growth Measurements

Cells were grown in YPD, diluted serially and spotted onto YPD + 200 mM $CaCl_2$ and incubated for 2 days at 30°C. *vph1Δ::KANMX4* deletion strain (Y07238) was obtained from Euroscarf.

Supplementary Table

Table 1: *In vitro* fluorescence of sfGFP and variants in *E. coli* cell free extracts

	Amino Acid										Rel. Fluorescence[1]
	**3**	**4**	**6**	**41**	**69**	**108**	**149**	**167**	**188**	**204**	**pH 5**
**PARENTAL**											
sfGFP	Lys	Gly	Glu	Lys	Gln	Thr	Asn	Ile	Ile	Gln	1.0
sfGFPmax		Arg									5.8
**ROUND 1**											
F1								Thr			3.8
A1			Val							His	4.4
C1										His	2.5
**ROUND 2**											
D5.1			Val		Leu					His	6.1
D5.2			Val	Asn		Ser	Tyr			His	9.3
**ROUND 3**											
A2		Arg					Tyr	Thr	Val	His	18.9
C1		Arg					Tyr			His	15.7
D1	Ile	Arg		Asn			Tyr	Thr		His	10.8

1. Relative fluorescence measured in cell-free extracts containing the respective sfGFP-variants compared to sfGFP. Fluorescence intensities were determined in 25 mM citrate-phosphate buffer with 5 mg/ml total protein. Note that the cell free extracts were not normalised for the amount of fluorescent protein.

Table 2: Overview of pH titration data

	pKa			Slope		
		2.5 %	97.5 %		2.5 %	97.5 %
**sfGFP**	5.9	5.7	6.0	-11.4	-12.7	-10.2
**quadruple**	4.5	4.4	4.6	-9.3	-10.4	-8.3
**N149Y/Q204H**	4.8	4.7	4.9	-10.1	-11.3	-9.0
**triple (167)**	4.3	4.2	4.4	-8.7	-10.0	-7.5
**triple (188)**	5.2	5.1	5.2	-8.9	-9.8	-8.0
**N149Y**	5.4	5.2	5.8	-5.6	-7.3	-4.1
**Q204H**	4.7	4.6	4.9	-6.8	-8.4	-5.4
**V206A**	5.8	5.7	5.9	-10.9	-12.2	-9.7
**tdGFP**	4.8	4.7	4.9	-9.2	-10.5	-8.0

Table 3: Overview of the Properties of Identified Variants

	**Location in protein**	**pH stability**	**Excitation spectra shift at low pH**	**Emission spectra shift at low pH**	**Oligomer formation**	**Protein Stability**	**Included in pH-tdGFP?**
sfGFP	-		Minor	Modest	No		Yes
N149Y	Dimer interface	Modest increase	No effect	Minor	Yes	Increase	Yes
Q204H	Dimer interface	Modest increase	No effect	Minor	Yes	Increase	Yes
I167T	Protein core	nd*	nd	nd	nd	nd	No
I188V	Protein core	nd	nd	nd	nd	nd	No
N149Y + Q204H	-	Increase	No effect	Minor	Yes	Increase	-
N149Y + Q204H + I167T	-	Increase	No effect	Major	Yes	Decrease	-
N149Y + Q204H + I188V	-	Increase	No effect	Minor	Yes	Decrease	-
N149Y + Q204H + I167T + I188V	-	Increase	No effect	Major	Yes	Decrease	-
pH-tdGFP	-	Increase	No effect	Very minor	No	Increase	-

* nd denotes not determined

Supplementary Figures

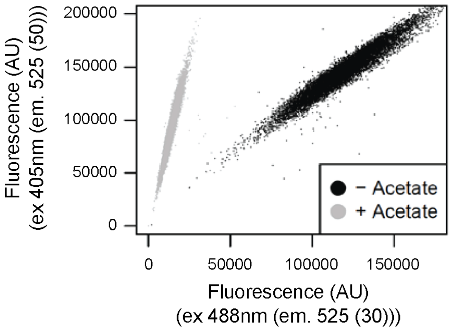

Supplementary Figure 1: Comparison of cells expressing sfGFP at high and low pH. *E. coli* cells expressing sfGFP were incubated in buffer with and without acetate and the ratio of fluorescence intensities of sfGFP at 405 nm and 488 nm with constant emission (525 nm) were compared. sfGFP excited at 488 nm converts from a highly fluorescent form at pH 7 (black) to a weakly fluorescent form at pH 5 (grey).

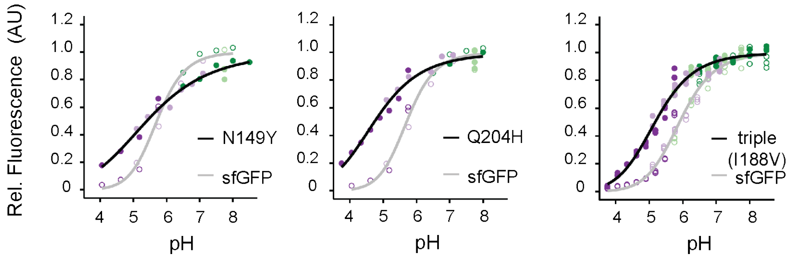

Supplementary Figure 2: Purified GFPs were diluted into buffers ranging in pH from 3.75 - 8.50 and fluorescence was measured (ex 488 nm, em 525 (5) nm). triple (I188V) denotes N149Y/Q204H/I188V. Lines represent the trend of the median fluorescence for the measured pH values. Filled and open circles indicated fluorescence values at each pH for the indicated variants and sfGFP respectively; colours indicate the buffer used (acetate (dark purple), MES (light purple), PIPES (light green), HEPES (dark green)). sfGFP curves on each graph are from the same date and plate as the indicated variant.

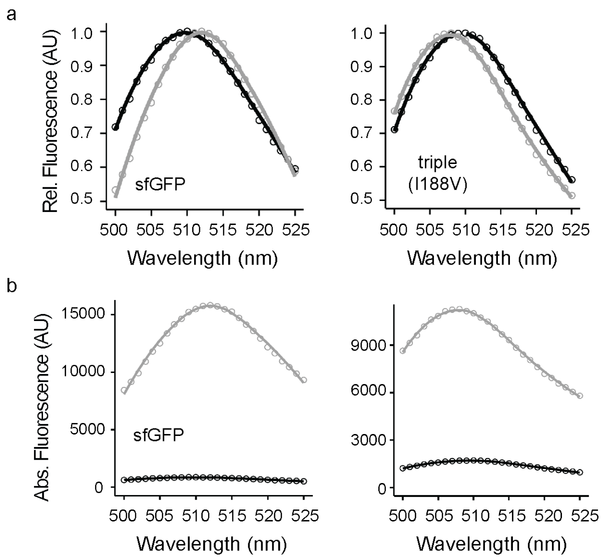

Supplementary Figure 3: (a) Normalized fluorescence emission scans (ex 488 nm) of indicated GFP variants at pH = 4.3 (black) and 7.5 (grey). triple (I188V) denotes N149Y/Q204H/I188V. (b) Raw data of a. Please note that the experiments were not performed on the same day and the reader settings were changed between experiments.

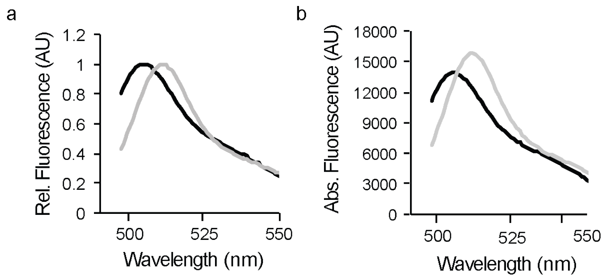

Supplementary Figure 4: (a) Fluorescence emission scans (ex 488 nm) of sfGFP (grey) and N149Y/Q204H/I167T (black) at pH = 7.5. (b) Raw data of a. Please note that the experiments were not performed on the same day and the reader settings were changed between experiments.

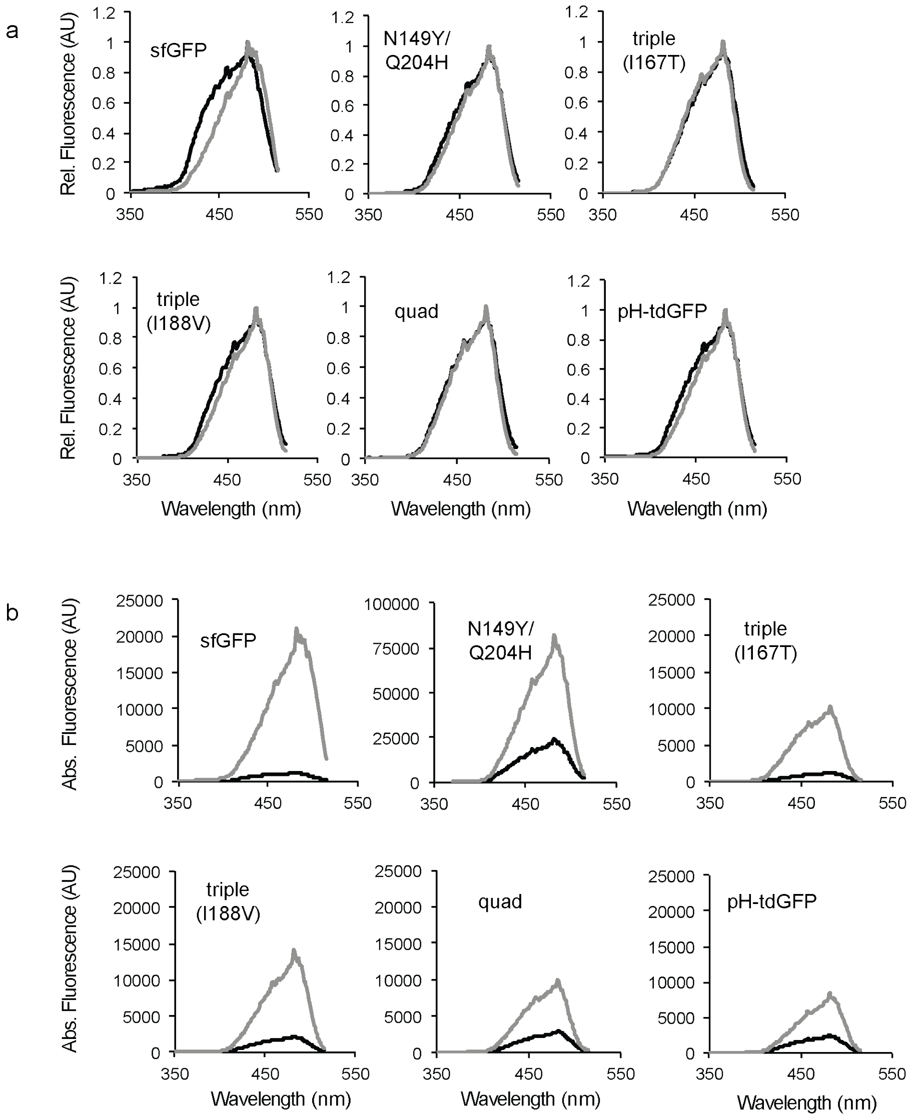

Supplementary Figure 5: (a) Fluorescence excitation scans (em 525 nm) of indicated GFP variants at pH = 4.3 (black) and 7.5 (grey). triple (I167T) denotes N149Y/Q204H/I167T; triple (I188V) denotes N149Y/Q204H/I188V; quad denotes N149Y/Q204H/I167T/I188V. (b) Raw data of a. Please note that the experiments were not performed on the same day and the reader settings were changed between experiments.

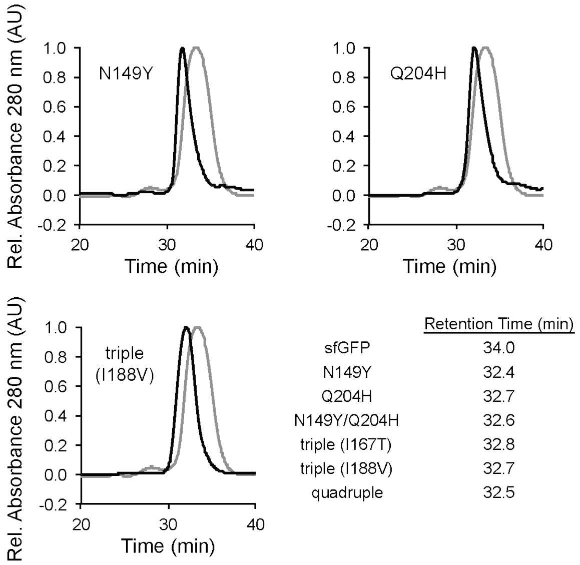

	Retention Time (min)
sfGFP	34.0
N149Y	32.4
Q204H	32.7
N149Y/Q204H	32.6
triple (I167T)	32.8
triple (I188V)	32.7
quadruple	32.5

Supplementary Figure 6: Oligomeric state of purified GFP variants was examined by size exclusion chromatography. Purified variants were separated with FPLC using Superdex S200 10/300 GL column. Retention time of the major peak is given in the table. triple (I167T) denotes N149Y/Q204H/I167T; triple (I188V) denotes N149Y/Q204H/I188V; quad denotes N149Y/Q204H/I167T/I188V. sfGFP is provided in grey as a reference.

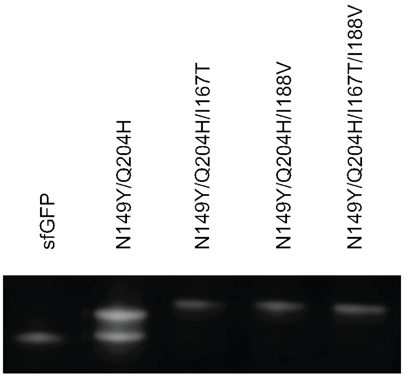

Supplementary Figure 7: Examination of the oligomeric state of the GFP variants. Purified proteins were subjected to semi-native SDS-PAGE and viewed using a blue light table (470 nm).

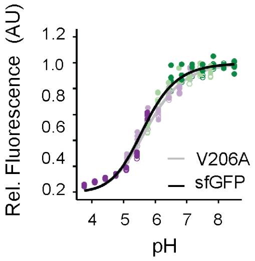

Supplementary Figure 8: Examination of the fluorescence of V206A at various pH. Purified V206A (black) were incubated in buffers ranging in pH from 3.75 to 8.5 and fluorescence intensity was measured. Lines represent the trend of the median fluorescence for the measured pH values. Filled and open circles indicated fluorescence values at each pH for V206A and sfGFP respectively; colours indicate the buffer used (acetate (dark purple), MES (light purple), PIPES (light green), HEPES (dark green)). sfGFP is provided in grey as a reference.

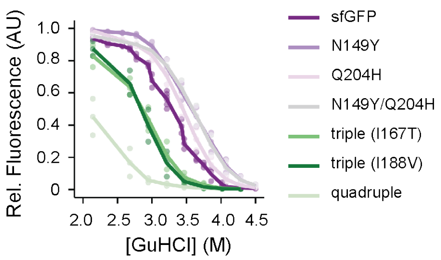

Supplementary Figure 9: Examination of the stability of pH-stable sfGFP variants to denaturant. Purified sfGFP or indicated variants were incubated in various concentrations of guanidinium hydrochloride, fluorescence was measured at steady state.

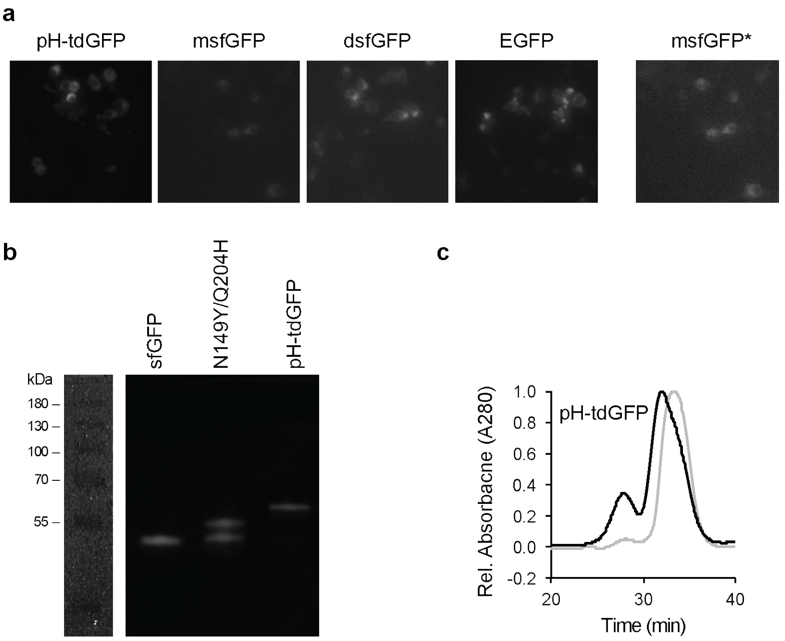

Supplementary Figure 10: Examination of the oligomeric state of pH-tdGFP. (a) Representative OSER assay images of HEK293 cells expressing the indicated fusions. Due to the lower brightness of msfGFP a second image with altered contrast is shown (msfGFP*). (b) Purified pH-tdGFP was subjected to semi-native SDS-PAGE and viewed using a blue light table (470 nm). The image was taken in a dark. The image was also taken in an illuminated room to visualise the size marker. (c) Purified pH-tdGFP was examined by FPLC size exclusion chromatography using Superdex S200 10/300 GL column. sfGFP is present as monomer and is shown as reference (grey line). Plots are scaled relative to the peak.

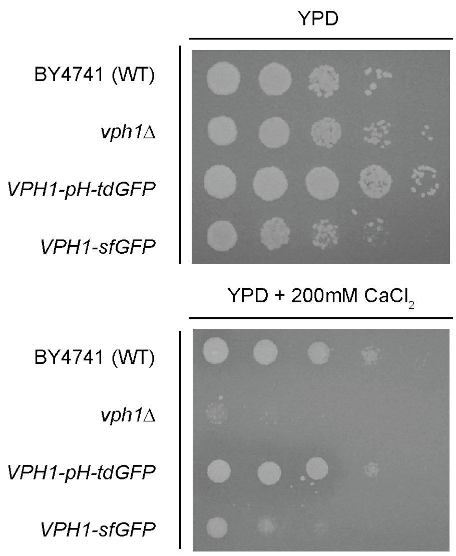

Supplementary Figure 11: Examination of the effect of the C-terminal pH-tdGFP or sfGFP tag on Vph1 function. Ten-fold serial dilutions of cells were spotted onto YPD or YPD containing 200 mM $CaCl_2$ and incubated at 30°C for 2 days. Equal numbers of cells were spotted at each dilution for each strain; cell number was estimated using OD600.

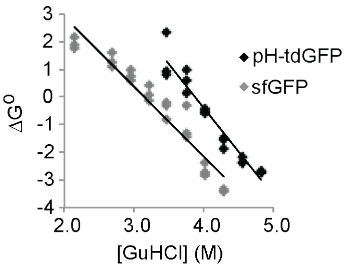

Variant	ΔG (kcal/mol)
sfGFP	7.96
tdGFP	12.29

Supplementary Figure 12: Examination of the stability of pH-tdGFP to denaturant. Purified sfGFP or pH-tdGFP were incubated in various concentrations of guanidinium hydrochloride, fluorescence was measured at steady state, and the ΔG of unfolding was estimated using the following equation $\Delta G^{\circ} = \Delta G(H_2O) - m[GuHCl]$.

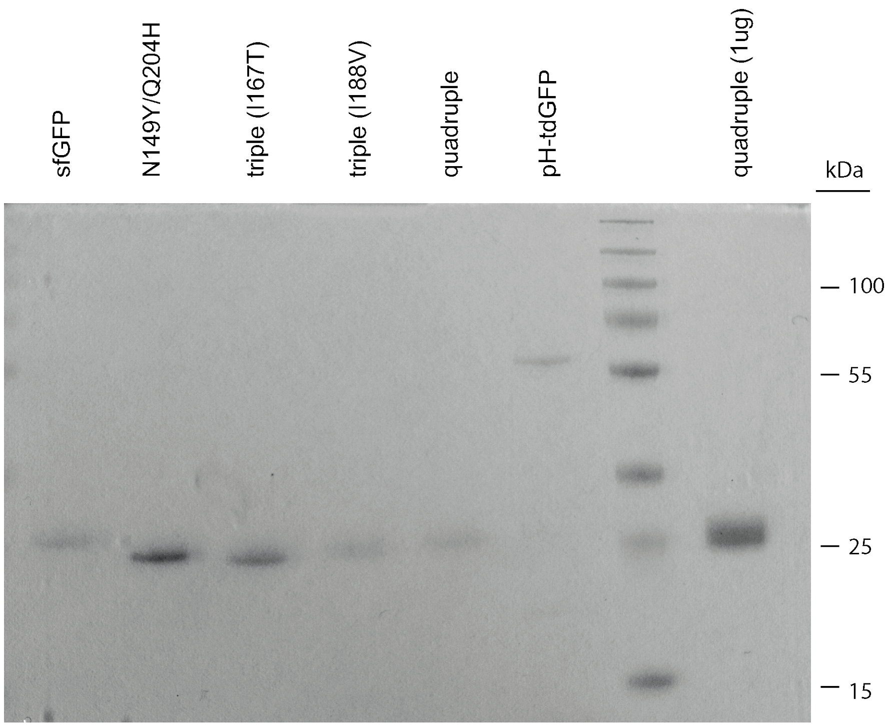

Supplementary Figure 13: SDS-PAGE of various purified proteins. Approximately 200 ng of each purified protein was added to 5x urea loading dye and boiled for 10 min at 95°C. N149Y/Q204H/I167T, N149Y/Q204H/I188V and N149Y/Q204H/I167T/I188V are denoted triple (I167T), triple (I188V) and quadruple, respectively. 1 μg of the quadruple mutant was included as a representative to indicate purity of protein preparations.

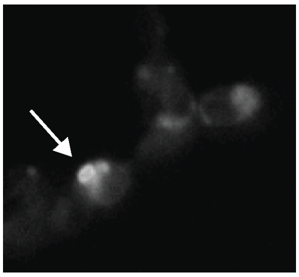

Supplementary Figure 14: Example of a cell with an OSER 'whorl' structure indicative of rearrangement of the endoplasmic reticulum. HEK293 cells transfected with OSER plasmids expressing GFP fusions to the C-terminal portion of cytochrome P450 were imaged 30 hours after transfection and cells were counted. The arrow indicates a cell counted as an abnormal cell containing a 'whorl'.
